# Clinical features of AIDS patients with Hodgkin’s lymphoma with isolated bone marrow involvement: report of 12 cases at a single institution

**DOI:** 10.7497/j.issn.2095-3941.2014.0024

**Published:** 2015-03

**Authors:** Marcelo Corti, Maria Villafañe, Gonzalo Minue, Ana Campitelli, Marina Narbaitz, Leonardo Gilardi

**Affiliations:** 1 HIV/AIDS Department, F. J. Muñiz Hospital, Buenos Aires 1406, Argentina; 2 Hematology Unit, F. J. Muñiz Hospital, Buenos Aires 1429, Argentina; 3 Histopathology Laboratory, F. J. Muñiz Hospital, Buenos Aires 1429, Argentina; 4 National Academy of Medicine, Histopathology Laboratory, Buenos Aires 1425, Argentina; 5 Sociedad Iberoamericana de Informacion Cientifica, Scientific Coordination, Buenos Aires 1061, Argentina

**Keywords:** Acquired immunodeficiency syndrome (AIDS), Hodgkin’s lymphoma (HL), bone marrow (BM)

## Abstract

**Objective:**

To study the main clinical and histopathological features of 12 patients with Hodgkin’s lymphoma (HL) diagnosed primarily from bone marrow (BM) involvement.

**Methods:**

We included 12 acquired immunodeficiency syndrome (AIDS) patients with HL assisted in the F. J. Muñiz Infectious Diseases Hospital since January 2002 to December 2013. The diagnosis of HL with primary BM involvement in patients was confirmed by clinical, histopathological, and immunohistochemical findings.

**Results:**

All patients presented “B” symptoms and pancytopenia. All of them had stage IV neoplasm disease because of BM infiltration. The median of CD4^+^ T-cell counts was 114 cells/μL, and mixed cellularity (MC) was the most frequent histopathological subtype of 92% cases.

**Conclusion:**

When other causes are excluded, BM biopsy should be performed in AIDS patients with “B” symptoms and pancytopenia to evaluate BM infiltration by atypical lymphocytes.

## Introduction

The risk of Hodgkin’s lymphoma (HL) is significantly increased in acquired immunodeficiency syndrome (AIDS) patients. Patients with human immunodeficiency virus (HIV) have a 5- to 15-fold relative risk compared with the general population. The most frequent subtypes of HL in HIV/AIDS patients are mixed cellularity (MC) and lymphocyte depletion (LD), whereas the nodular sclerosis (NS) subtype predominates in the general population^1,2^. Primary extranodal presentation of HL is rare and includes less than 0.25% of patients^3^. Moreover, the incidence of bone marrow (BM) involvement in HL varies with the histological subtype. In this paper, we report a group of 12 patients with HL associated with HIV infection diagnosed primarily from BM involvement.

## Materials and methods

We retrospectively reviewed the records of patients in the F. J. Muñiz Infectious Diseases Hospital, a reference hospital for infectious diseases, to search for all the HIV-positive patients with lymphoma with associated confirmation of the case by histological examination. Only adult patients who were hospitalized between 2002 and 2013 were considered. The inclusion criterion for this specific analysis was the diagnosis of HL with isolated BM involvement. Data on demographic and clinical profiles, HIV characteristics, laboratory profiles including lactate dehydrogenase (LDH) levels, CD4^+^ T-cell count, presenting symptoms at the time of neoplasm diagnosis, and hepatitis C virus (HCV) serological status were recorded. This study was approved by the ethics committee at the F. J. Muñiz Infectious Diseases Hospital.

All the patients underwent chest X-ray and complete tomography scan of head, neck, thorax, abdomen, and pelvis with oral and intravenous contrast as part of the initial staging work-up. Diagnosis was confirmed by histopathological examination of the trephine BM biopsy performed on the posterior iliac crest (unilateral). The core of BM biopsy was fixed in Bouin’s solution. Afterward, all smears were decalcified using an EDTA-based solution, embedded in paraffin, and then sectioned. To detect the presence of opportunistic infections, all smears were stained in hematoxylin and eosin, Giemsa, PAS, Ziehl-Neelsen, and Grocott methenamine silver stain. HL diagnosis was confirmed through the identification of classical Reed-Sternberg (R-S) cells or their variants in an adequate cellular background and was made according to the World Health Organization classification of hematological malignancies. Immunohistochemical stains with CD30, CD15, leukocyte common antigen (LCA), CD45, CD20, and CD3 antibodies were obtained from DAKO Diagnosis (Denmark). The presence of Epstein-Barr virus (EBV)-associated latent membrane protein-1 (LMP-1) by immunohistochemistry was analyzed in BM sections.

### Statistical analysis

All data were tabulated using Microsoft Excel^®^ 2007. A descriptive statistical analysis was performed. Measures of central tendency and variability were calculated. When possible, Student *t*-test and correlation tests were completed. All statistical tests were performed using SPSS^®^ 22.0.

## Results

Between January 2002 to December 2013, 115 HIV-positive patients with lymphoma were identified, and 38 of these patients had HL (33%). Of the 38 patients with HL, 12 had isolated BM involvement without adenopathy or other solid organ infiltration. Thus, 12 patients with isolated BM involvement were included in our descriptive analysis.

During the period of the study, we performed 429 BM biopsies; 12 (2.7%) had diagnosis of HL with primarily and isolated BM involvement. Most patients were men (*n*=11; 92%). The mean age was 35.91±8.69 years (range, 26-51 years), with a nearly normal distribution. Intravenous drug use (IVDU) was the main HIV infection risk factor (66.6%). Median time from diagnosis of HIV infection to HL was 10 years. All patients presented “B” symptoms, such as prolonged and unexplained fever, weight loss (median 5 kg), night sweats, and pancytopenia. No evidence of concomitant opportunistic infection was found in the microbiological analysis.

Pancytopenia was demonstrated in all patients. Median red cell count was 2.2×10^6^ cells/µL (range, 1.89-3.54 ×10^6^ cells/µL). Median values of hemoglobin levels and hematocrit were 6 g/dL (range, 5.5-10.9 g/dL) and 20% (range, 17%-34%), respectively. White cell count was below normal values in all cases (median count: 3,100 cells/µL, range, 1,100-3,700 cells/µL). Platelet count was abnormal in almost all patients (median count: 94,000 cells/µL; range, 41,000-189,000 cells/µL).

The median CD4^+^ T-cell count was 114 cells/µL (range, 17 to 282 cells/μL) with significant heterogeneity among individual patients. In only two subjects, CD4^+^ T-cell count was above 200 cells/µL. Eight patients were also infected with HCV, and all of them had a history of IVDU. Initial values of LDH were available for six patients and were elevated in four of them (normal value up to 350 U/L). Computed tomography scans of chest, abdomen, and pelvis demonstrated negative results for lymphadenopathies and liver or spleen involvement. No gallium or positron emission tomography (PET) scans were performed. Demographic, clinical, and histopathological findings are listed in **Table 1**.

**Table 1:** Demographic, clinical, and histopathological findings in 12 patients with HL diagnosed primarily in BM

Patient	Sex	Age (years)	Risk factor	CD4	HCV	LDH
#1	M	26	IVDU	ND	Pos	ND
#2	F	29	IVDU	ND	Pos	ND
#3	M	27	IVDU	195	Pos	ND
#4	M	34	IVDU	17	Pos	ND
#5	M	26	IVDU	39	Pos	ND
#6	M	31	IVDU	123	Pos	ND
#7	M	49	UHSC	52	Neg	600
#8	M	40	IVDU	56	Pos	796
#9	M	35	UHSC	104	Neg	318
#10	M	41	UHSC	237	ND	390
#11	M	51	UHSC	282	Neg	638
#12	M	42	IVDU	198	Pos	360

M, male; F, female; HCV, hepatitis C virus; LDH, lactate dehydrogenase; IVDU, intravenous drug user; UHSC, unprotected heterosexual contact; Pos, positive; Neg, negative; ND, no data.

**Figure 1:**
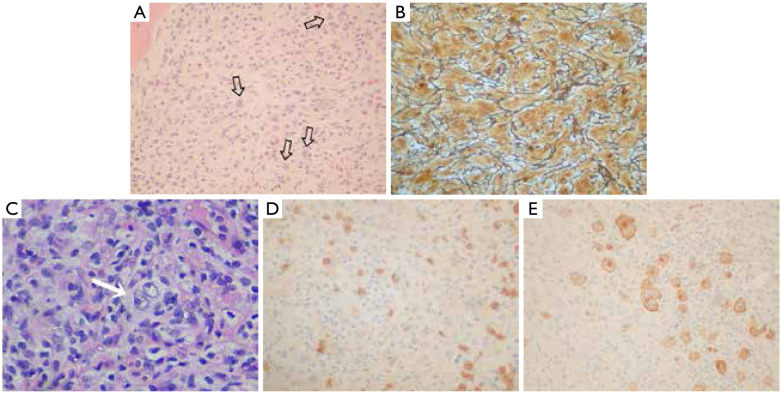
(A) Diffuse involvement of bone marrow by HL with the identification of Reed-Sternberg cells (arrows) in an appropriate background (H&E, ×10); (B) Diffuse bone marrow fibrosis observed with methenamine-silver stain (×100); (C) Focal pattern of BM involvement in HL with the presence of a large binucleated Reed-Sternberg cell (arrow) (H&E, ×100); (D) Immunohistochemistry show some CD15^+^ Reed-Sternberg cells (IHC,×40); (E) Immunohistochemistry with expression of CD30 antigen in an adequate cellular background by Reed-Sternberg cells (IHC, ×100).

All cases presented the minimum requirement of HL diagnosis, including identification of the classical R-S cells or their variants in an adequate cellular background (**Figure 1A,B**). The type and extent of BM involvement, the percent of biopsy involved, the type of infiltration pattern, the immunostaining reactivity of R-S cells, the characteristics of the accompanying lymphocytes, and the EBV-associated LMP-1 results in patients in which this information were available are described in **Table 2**.

**Table 2:** Histological features in 12 patients with Hodgkin’s lymphoma diagnosed primarily in the bone marrow

Patient	Infiltration pattern	Infiltration (%)	Reed Sternberg cells				Background lymphocytes
			CD30	CD15	CD20	LMP1	
#1	NDA	NDA	NDA	NDA	NDA	NDA	NDA
#2	NDA	NDA	NDA	NDA	NDA	NDA	NDA
#3	NDA	NDA	NDA	NDA	NDA	NDA	NDA
#4	Nodular	40	Positive	Positive	Negative	Negative	CD3
#5	Diffuse	100	Positive	Negative	Negative	NP	CD3
#6	Nodular	40	Positive	Positive	Negative	NP	CD3
#7	Nodular	35	Positive	Positive	Negative	NP	CD3
#8	Diffuse	80	Positive	Positive	Negative	NP	CD3
#9	Diffuse	90	Positive	Positive	Negative	Positive	CD3
#10	Nodular	40	Positive	Positive	Negative	NP	CD3
#11	Diffuse	80	Positive	Positive	Negative	Positive (weak)	CD3
#12	Diffuse	50	Positive	Positive	Negative	NP	CD3

NDA, no data available; NP, not performed.

## Discussion

HL is the most common non-AIDS-defining malignancy in patients infected with HIV. BM infiltration is rare as the initial or the only site of involvement in HL. In the general population, the involvement of the BM ranges from 2% to 32%, with an average of 10%^4^. In addition, the incidence of BM involvement is less than 1% in patients with early stage of the disease^5^.

In a large group of patients with BM involvement associated with HL, the incidence was 4.8% in all stages of neoplasm disease. When the authors included only the patients with stage IV neoplasm disease, 32% of them had BM infiltration^5^.

Trephine biopsy is the best method to evaluate BM involvement in HL. The aspirate is not indicated for the detection of BM involvement in HL because of the elevated number of false negative cases, which is probably due to the focal infiltration pattern and the accompanying fibrosis^6^. The infiltration of BM by HL may indicate both the vascular dissemination of the disease and/or a primary BM-HL that originated from BM. BM involvement varies according to the histological subtype: 10% in classical MC, 1% in LP, and 3% in NS subtype. LD is the rarest subtype that includes the involvement of BM in clinical presentation^7–9^.

A few cases of HL diagnosed primarily from BM biopsy have been reported in the literature^10^. The infiltration of BM as the initial site of the disease is more common in HIV patients compared with the general population. Diagnosis of HL in BM biopsy is more frequent in HIV-seropositive patients with stage IV of the disease^11^. Ponzoni *et al*.^12^ described only six patients with BM involvement as the only site involvement at the diagnosis of the neoplasm disease in a group of 42 HIV patients with HL.

In HIV patients, BM biopsy is performed in subjects with pancytopenia or prolonged fever of unknown origin. Ponzoni *et al*.^12^ reported that all their patients presented fever, blood cytopenias, and severe CD4-T LD, which we also observed in our patients. In our experience, BM biopsy is also useful in patients with B symptoms in which HL is not suspected. In these patients, disseminated tuberculosis, atypical mycobacteria, and systemic mycosis are often suspected clinically. The presence of nonspecific B symptoms may conceal an infectious disease, although the patients have a lymphoma. An elevated number of AIDS patients have B symptoms and pancytopenia, and BM biopsy should be performed when other etiologies are excluded.

The presence of R-S cells in the histopathological study of BM biopsy is definitive and confirmative on the diagnosis of BM infiltration. Moreover, other patterns of BM infiltration by HL include a global hypocellularity with “scattered” cells, fibrosis (**Figure 1B**), and necrosis^13,14^.

The pattern of infiltration can be diffused or focal. Diffused infiltration is more frequent and includes extensive areas between bone trabeculae (**Figure 1A,B**). The focal pattern is less frequent and shows small or nodular patches that surround the normal tissue (**Figure 1C**)^13,15^.

R-S cells have a characteristic immunohistochemical profile that can be seen in BM biopsy. In addition, the immunohistochemical study of BM biopsy in patients with HL is very useful to complete diagnostic information. With adequate immunohistochemical studies, the diagnosis of HL can be obtained in the majority of cases. The typical immunophenotypic pattern of classic HL includes the expression of CD15 and CD30 (**Figure 1D,E**) and the negativity of LCA, CD20, and CD3 antibodies^16^.

The histological subclassification of HL on BM alone includes some discrepancies between lymph nodes and BM histology^17^. Classic and nodular LP HL could be easily differentiated based on morphological and immunohistochemical studies^18^. The presence of tissue eosinophilia seems to be an important morphological feature, and the quantification of eosinophils should be useful in pathological description in patients with classic HL^19,20^. The differential diagnosis of BM involvement in HL includes peripheral T-cell lymphomas and ALK lymphoma. Other differential diagnoses that should be considered are metastatic carcinoma, chronic lymphocytic leukemia in Ritcher transformation, and immune disorders including HIV infection^21^.

Recent studies suggest that PET is an alternative method to detect BM involvement in HL^22^. However, false negative cases of BM involvement in HL have been reported using PET scan^23,24^. For this reason, BM biopsy is the gold standard to detect BM infiltration in HL^15^.

The gold standard therapy for HL associated with AIDS is the combination of highly active antiretroviral therapy (HAART) plus chemotherapy. The introduction of HAART improved the prognosis for these patients. Before the HAART era, the median survival of patients with HIV-HL was less than 20 months. In the post-HAART era, Hentrich *et al*.^16^ demonstrated significantly better 2-year overall survival in patients receiving HAART plus chemotherapy compared with those not receiving HAART (74% *vs*. 30%, *P*<0.001). Complete remission and HAART response were significantly associated with better survival.

This study is a single hospital analysis of HL primary diagnosis in BM in HIV-seropositive patients, so our findings should not be generalized. Several strengths of our groups should be mentioned, such as the multidisciplinary approach by oncologists, pathologists, and infectologists in a highly specialized hospital. The limitations of this study include its retrospective nature, the absence of a follow up, the small size of the cohort, and the characteristics of a single center of infectious diseases. The small size of our cohort, including some patients of the pre-HAART era, represents a limitation in evaluating the impact of HAART plus chemotherapy in the survival of these patients. However, to the best of our knowledge, this is the largest series of HL in AIDS patients with isolated BM involvement.

In conclusion, BM may be the only or the initial site of HL in AIDS patients. Having a high index of suspicion in these patients and performing a BM trephine biopsy to observe the changes of Hodgkin’s lesions in the BM, especially in patients with pancytopenia or systemic B symptoms, are necessary.
